# Evaluation of the Safe Water Optimization Tool to Provide Evidence-Based Chlorination Targets in Surface Waters: Lessons from a Refugee Setting in Uganda

**DOI:** 10.1021/acs.est.4c04240

**Published:** 2024-10-09

**Authors:** Camille Heylen, Gabrielle String, Doreen Naliyongo, Syed Imran Ali, James Brown, Michael De Santi, Vincent Ogira, Jean-François Fesselet, James Orbinski, Daniele Lantagne

**Affiliations:** † Civil and Environmental Engineering, Tufts University, Medford, Massachusetts 02155, United States; ‡ Civil and Environmental Engineering, Lehigh University, Bethlehem, Pennsylvania 18015, United States; § Oxfam, Kampala, P.O. Box 6228, Uganda; ∥ Dahdaleh Institute for Global Health Research, York University, Toronto, Ontario M3J 2S5, Canada; ⊥ Médecins Sans Frontières, Amsterdam 1018 DD, The Netherlands; # Friedman School of Nutrition, Tufts University, Boston, Massachusetts 02111, United States

**Keywords:** chlorine taste and odor, disinfection by-products, humanitarian crisis, microbiological water quality, user acceptability, water system operators, water, sanitation, and hygiene

## Abstract

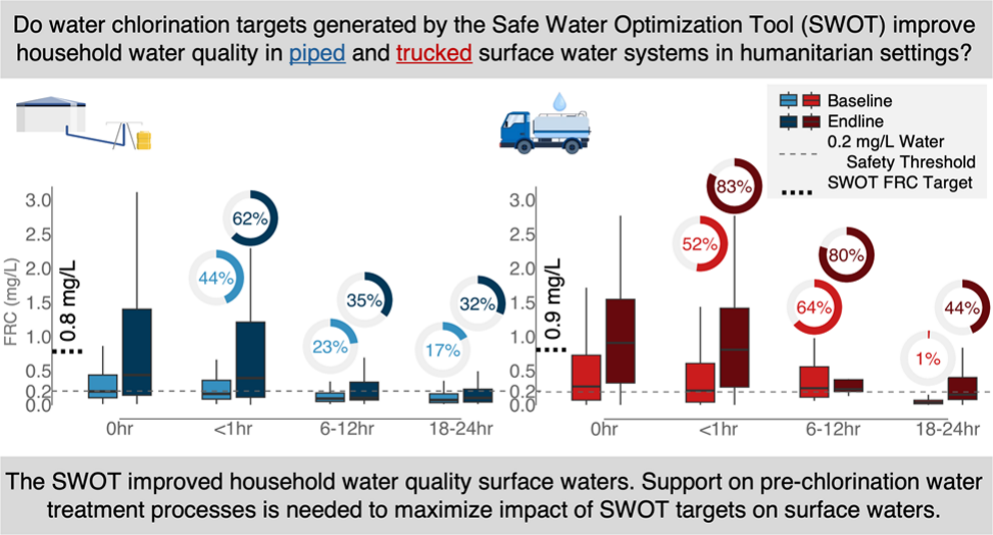

The Safe Water Optimization Tool (SWOT) generates evidence-based point-of-distribution free residual chlorine (FRC) targets to adjust chlorine dosing by operators and ensure water quality at point-of-consumption. To investigate SWOT effectiveness in surface waters, we conducted two before-and-after mixed-method evaluations in a Uganda refugee settlement served by piped and trucked surface water systems. We surveyed 888 users on water knowledge, attitudes, and practices; collected 2768 water samples to evaluate FRC,*Escherichia coli*, and disinfection by-products (DBPs) concentrations; and conducted nine key-informant interviews with system operators about SWOT implementation. After baseline data collection, SWOT chlorination targets were generated, increasing point-of-distribution FRC targets from 0.2 to 0.7–0.8 mg/L and from 0.3 to 0.9 mg/L for piped and trucked systems, respectively. At endline, household point-of-consumption FRC ≥ 0.2 mg/L increased from 23 to 35% and from 8 to 42% in the two systems. With these increases, we did not observe increased chlorinated water rejection or DBPs concentrations exceeding international guidelines. Informants reported that SWOT implementation increased knowledge and capacity and improved operations. Overall, SWOT-generated chlorination targets increased chlorine dosage, which improved household water quality in surface waters although less than previously documented with groundwater sources. Additional operator support on prechlorination water treatment processes is needed to ensure maximally effective SWOT implementation for surface water sources.

## Abstract 2

This work fills gaps on Safe Water Optimization Tool implementation in systems reliant on surface waters to ensure water quality at the point-of-consumption.

## Introduction

Access to safe drinking water, sanitation, and hygiene (WASH) is a universal human right, essential for the survival and dignity of people, and critical to infectious disease control in humanitarian emergencies.^1^ In humanitarian contexts, chlorination is the water treatment method most commonly recommended because it effectively inactivates most pathogens that cause diarrheal diseases and provides residual protection against recontamination.^2^ International water treatment guidelines recommend a fixed free residual chlorine (FRC) of 0.2–0.5 mg/L, with pH < 8 and turbidity < 5 NTU, at water distribution points (e.g., public tapstands)^1,3^ to protect stored water against microbiological recontamination at point-of-consumption (e.g., households). However, this FRC target does not account for chlorine decay during collection, transport, and household storage^4^ between the point-of-distribution and the point-of-consumption and previous studies have shown that these guidelines fail to reliably provide adequate FRC concentrations at point-of-consumption.

Thus, it is recommended to optimize point-of-distribution chlorination targets to ensure adequate point-of-consumption FRC.^1,2,4^ The Safe Water Optimization Tool (SWOT) was developed to generate site-specific point-of-distribution FRC targets that optimize the proportion of households with sufficient FRC at the point-of-consumption for the typical duration of water storage. A proof-of-concept implementation of the SWOT conducted at a refugee settlement in Cox’s Bazar (Bangladesh) generated a point-of-distribution FRC target of 0.8 mg/L.^5^ When SWOT targets were achieved at tapstands, 85% of households had FRC ≥ 0.2 mg/L at 15 h postdistribution, compared to 43% when international guidelines were achieved at tapstands. The SWOT has also been used in refugee camps in South Sudan, Jordan, and Rwanda where point-of-consumption FRC concentrations ≥ 0.2 mg/L of 71, 82, and 68% were reached, respectively.^6^

To date, SWOT implementation studies have primarily been in settings with groundwater supplies, which require less pretreatment before chlorination than surface water supplies.^1^ Given the range of water sources encountered in emergency settings, there is a research gap on whether SWOT can optimize water chlorination and ensure sufficient household FRC in systems reliant on surface water.

FRC optimization often entails increasing point-of-distribution chlorination targets, which present two concerns. The first is that increased taste and odor (T&O) can hinder the use of chlorinated water by the population who may seek alternate, less-safe sources for drinking water.^7−9^ Thus, it is essential to balance increasing point-of-distribution FRC targets with population-specific T&O rejection thresholds. Second, disinfection by-products (DBPs) concentrations are linked to potentially carcinogenic properties^10−12^ and DBPs increase with concentrations of organic precursors, chlorine dosage, storage time, temperature, and pH. These factors are often poorly controlled in emergency water systems, and point-of-consumption DBPs concentrations should be below international standards.^3^

To broaden the SWOT evidence base, we evaluated SWOT implementations in a Ugandan refugee settlement served by surface water. The objectives of our mixed-methods evaluation were to (1) evaluate SWOT effectiveness in ensuring point-of-consumption water quality in systems using surface water; (2) understand and integrate affected population chlorine T&O rejection thresholds; (3) characterize DBPs concentrations in chlorinated surface water samples at baseline and endline; and (4) understand water system operator experience during SWOT implementation. This evidence will benefit the WASH sector by helping develop generalizable tools for improving the quality of distributed water, community engagement and accountability, and public health risk reduction.

## Methods

**Figure 1:**
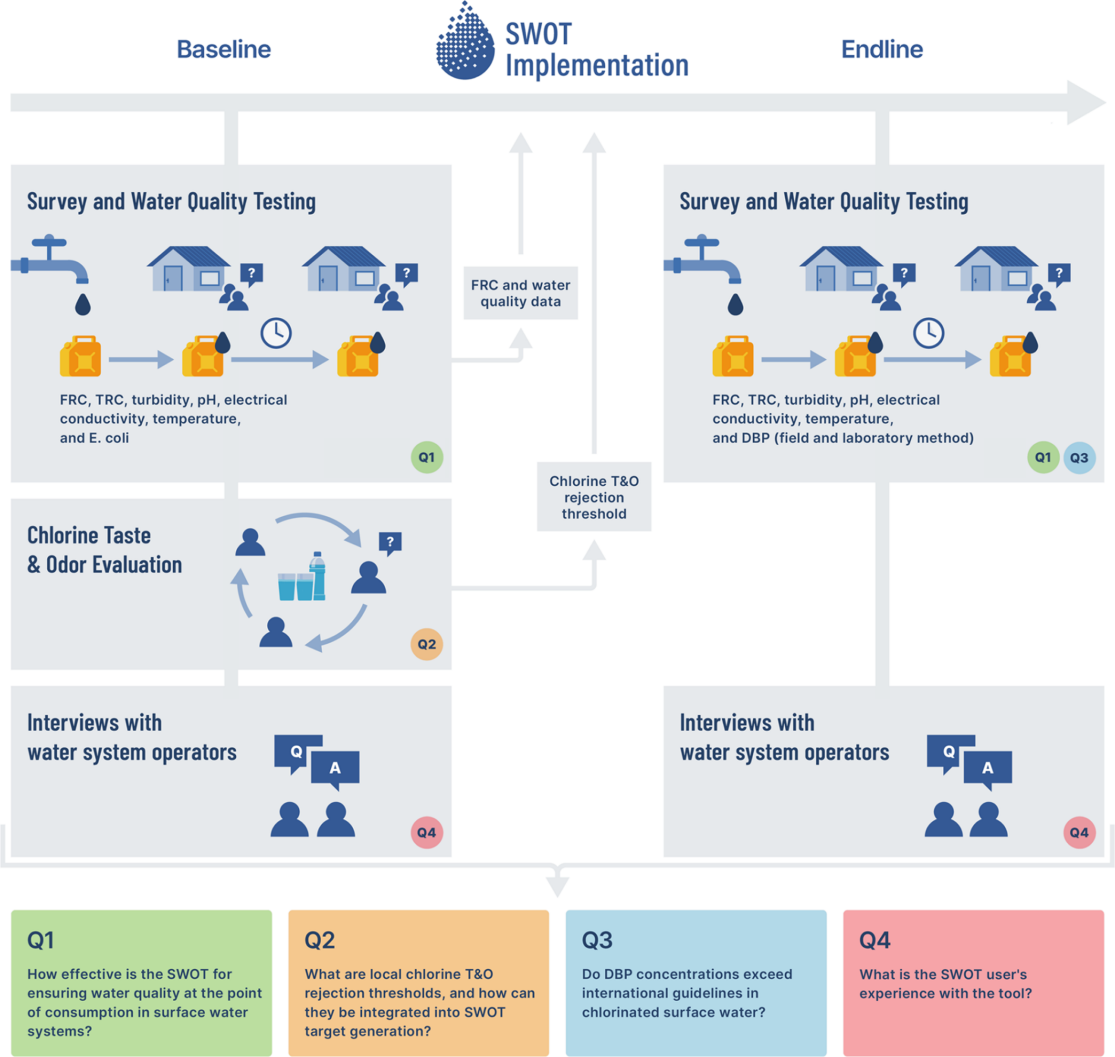
Study design for mixed-methods evaluation of the SWOT at the Kyaka II refugee settlement in Uganda. Free residual chlorine (FRC), total residual chlorine (TRC), and disinfection by-products (DBPs) were tested at baseline and/or endline. Chlorine taste and odor (T&O) evaluations were conducted at baseline. SWOT blue digitized water drop logo provided by Syed Imran Ali, Safe Water Optimization Tool Lead.

We conducted two mixed-methods evaluations at the Kyaka II refugee settlement in Uganda, including (1) surveying water users and collecting water quality data at point-of-distribution and point-of-consumption; (2) conducting chlorine T&O acceptability evaluations and DBPs testing and integrating those results into SWOT target development; and (3) interviewing water system operators on experience using the SWOT (Figure 1). Data were collected at baseline (before SWOT implementation), which were also used to generate SWOT FRC targets, and at endline (after SWOT implementation).

### Site Background

In collaboration with Oxfam Uganda, two water systems in Kyaka II were selected for study inclusion because of their use of surface water and different distribution system types. Both systems used surface water from the Sweswe Dam water treatment plant. At the Sweswe plant, surface water was pumped from the dam to an aerator for iron removal, then to settling tanks with coagulant for sedimentation, then to holding tanks, and last to either one of three reservoirs serving three different piped distribution systems or directly to water trucks.

One of the three piped systems was included in this study, serving the zones of Sweswe and Itambabiniga in Kyaka II. Chlorine solution made using high-test calcium hypochlorite (HTH) was added during reservoir filling to enhance mixing. After 30–60 min of contact time, water was released through 17 km of distribution lines to tapstands. This process was repeated twice daily. In the water trucking system, water from a holding tank was pumped into tanker trucks and chlorine solution was added. During the study, the trucks left 15–30 min after chlorination and traveled 15–45 min to distribution tanks, providing 30–60 min of contact time. Oxfam aimed for 0.5 mg/L FRC in point-of-distribution water in Kyaka II; however, during our study, there were no treatment, point-of-distribution, or point-of-consumption level FRC monitoring data available.

### Ethics Approvals

Study protocols were approved by the Tufts University Institutional Review Board (STUDY00001674), the United Nations High Commission for Refugees office in Kyaka II, and the Office of the Prime Minister’s Department for Refugees in Kampala, Uganda. A local research team was assembled, consisting of a research manager, data collectors, and members of village health teams (VHT). For community survey participants, a data collector was paired with a VHT who helped translate consent forms and survey questions from English or Swahili to local languages. All local team members were trained in person by a Tufts University team member on the ethical conduct of research, including how to randomly select participants at the point-of-distribution, obtain consent, deliver questions, record answers, and prevent bias. Verbal consent was obtained from all participants before the beginning of data collection.

### Surveys and Water Quality Testing

#### Sample Size Calculation

Based on an expected minimum 0.05 mg/L point-of-consumption FRC concentration increase, a sample size of 204 provides 80% power to detect a difference in before-and-after SWOT implementation FRC concentrations at 95% confidence. With an estimated 10% attrition adjustment, the sample size was 225 participants per group. Therefore, we intended to recruit 900 participants (225 for each system (piped and trucked) and period (baseline and endline)) and conduct 1800 surveys (2 visits (initial and follow-up) per participant).

#### Baseline Initial Survey

Potential adult participants were approached while collecting water from tapstands and tanks and asked if they would like to participate. If they consented, a water sample was collected from the point-of-distribution at the same time the participant collected water. Participants were then accompanied to their homes by data collectors for an initial survey that consisted of 23 observations and 67 questions on household demographics and water-related knowledge, attitudes, and practices. Answers were recorded using KoboToolbox (Cambridge, MA) on tablets. At the end of the survey, participants were asked to provide a cup of drinking water. These samples were analyzed on-site for temperature, pH, and electrical conductivity (EC) using a PC60 probe (Apera Instruments, Columbus, OH), free and total residual chlorine (FRC and TRC) using either a Palintest Lumiso (Tyne and Wear, U.K.) or a Lamotte 1200 DPD meter (Lamotte, Chestertown, MD), and turbidity using either the Lumiso or a Lamotte 2020we turbidimeter. All probes and meters were calibrated daily.

#### Baseline Follow-Up Survey

Households were revisited 3–24 h after the initial survey. The visit time depended on the longest typical duration of household water storage and logistical constraints in accessing the settlement. Follow-up surveys were conducted if water collected during the initial visit was still available (ensured by a mark left on the water container and survey questions) and consisted of 10 observations and 26 questions on use of that water (Annexes S1 and S2). At the end of the survey, participants were again asked to provide a cup of drinking water, and the same water quality analyses were conducted.

#### Baseline Microbiological Water Quality Tests

Paired point-of-consumption water samples at initial (*T*_0_) and follow-up (*T*_3–24 h_) visits were collected from 10 random households for *E. coli* analysis in both water systems. A 118 mL sample of water was aseptically collected into sterile WhirlPak bags with sodium thiosulfate (Nasco, Fort Atkinson, WI), placed on ice, and transported to a field laboratory for microbiological analysis within 8 h of sample collection. Samples were tested using an Aquagenx Compartment Bag test kit (Chapel Hill, NC) following standard directions. Results were recorded in most probable number (MPN)/100 mL, with 10% duplicates and 5% blanks processed for quality control.

#### SWOT Implementation

After baseline data collection, water supply operators in both systems were trained by York University staff to use the SWOT to generate point-of-distribution FRC targets^13^ that would protect household stored water for the typical duration of household storage and use. Additionally, the SWOT team used population-specific chlorine T&O data to ensure targets were within T&O acceptability limits. Please note chlorine T&O evaluations were conducted before SWOT implementation as part of this research, using ASTM E679-04 Forced-Choice Triangle Test^14^ and Standard Method 2160 Flavor Rating Assessment,^15^ like other papers.^16^ Methods and Results are described in a separate paper.^17^ Upon request, York University staff also provided training to water system operators on the Modified Horrock’s Test^18^ to modify chlorine dosage levels and provided advice on how to adjust chlorination to meet SWOT targets (including by monitoring/increasing the chlorine solution strength or by increasing the volume of chlorine solution added).

#### Endline Initial and Follow-Up Surveys

These were conducted in the same manner as at the baseline.

#### Endline DBPs Tests

At the endline, 20 paired point-of-distribution and point-of-consumption (*T*_3–24 h_) water samples were randomly collected from piped system households to test DBPs using two methods: the Hach THM Plus Method 10132 and a gold standard method (USEPA Method 8260C) at a certified laboratory (α Analytical, Westborough, MA). Samples were collected as described in method protocols, stored on ice, and stored in a 4 °C refrigerator. Vials for on-site processing were stored up to 3 days. For quality control, one blank and one standard were processed for each on-site batch. Laboratory vials were packed on ice and shipped to Boston, MA, for processing within 14 days of collection. Please note samples were not acidified before shipping.

The Hach method provides one cumulative result for the four regulated trihalomethanes (THMs) including chloroform, bromodichloromethane, dibromochloromethane, and bromoform in ppb and also includes compounds that interfere with the test. The laboratory provided individual analyte results for each of the regulated THMs. Laboratory results were summed to facilitate comparison. A quantile regression model was developed to predict the upper 95th percentile of THMs concentration based on FRC concentrations and worst-case water quality parameters linked to the greatest THMs production (including water temperature, EC, turbidity, and pH).

#### Data Analysis

Data were uploaded to KoboToolbox by the local research team and reviewed regularly by Tufts University staff. Data points were dropped if FRC or sampling times were erroneous between initial and follow-up visits (e.g., greater FRC at follow-up than initial). Frequency tables and descriptive statistics were developed to summarize household demographics, WASH conditions and behaviors, and water quality variables by the water system. Turbidity, pH, FRC, and THMs results were compared to WHO guideline values. Analytical statistics were used to compare FRC concentrations before and after SWOT implementation using Wilcoxon rank-sum tests. To assess associations between household demographics, WASH conditions and behaviors, and water quality, Chi-square and Wilcoxon rank-sum tests were used for categorical and continuous parameters, respectively. All *p*-values were two-tailed and statistical significance was set at *p* < 0.05 with 95% confidence intervals. Data were analyzed in RStudio (Posit, Boston, MA).

### Interviews with Water System Operators

Interviews were conducted at baseline and endline with water system operators to understand their background and training; opinions of the Kyaka II water supply and treatment program; experience implementing and using the SWOT; and suggestions for improvement. Interviews were conducted on-site with a digital recorder or via Zoom with online recording. Interviews were translated (if necessary), transcribed, and coded using NVivo (Burlington, MA), and the results are presented herein using emergent themes.

## Results

Overall, we surveyed 888 water users (439 piped systems, 449 trucked); collected 2768 water samples for analysis (Table S3); and conducted nine water system operator interviews. First, we present the piped and trucked system evaluations, followed by interview results.

### Piped System Evaluation

#### Site and Population

All 17 public tapstands serving the Sweswe and Itambabiniga zones were included in baseline and endline study phases (map available in Figure S1). Overall, 216 and 223 participants were enrolled at baseline and endline, respectively. Baseline and endline populations were largely similar in terms of water transport and storage practices, with some differences described herein that may influence chlorine decay (Table S1). Most participants reported collecting water in opaque 20 L jerrycans with small openings, with baseline participants more likely to collect water in >1 container (*p* < 0.001), and endline participants more likely to have a covered container (*p* = 0.003). Participants reported mostly collecting water in mornings and evenings, and some participants did not always collect tapstand water because of lack of running water and crowding (16% baseline, 5% endline, *p* < 0.001). Overall, 68% of people at baseline and 58% at endline stored water for <12 h, and 93 and 100% stored water for <24 h, respectively (*p* < 0.001).

#### Baseline

Data were collected over 15 days during April–May 2022. In total, 174 (of 216 collected) paired water quality measurements were included in the analysis. Data points were removed due to missing data (*n* = 27) and when FRC was higher at follow-up than at point-of-distribution (*n* = 15), which could be due to households inaccurately reporting household treatment. The median FRC concentration was 0.20 mg/L at point-of-distribution, with high variability (range 0.01–3.8 mg/L) (Table 1). Overall, 50% of point-of-distribution water samples had FRC < 0.2 mg/L, not meeting minimum standards. While the pH was within the range of 5–7.6 for effective chlorination (<8 recommended), the turbidity (1.1–59.1 NTU, 11.1 NTU median) was not (<5 NTU recommended).^3^ Water temperature during baseline was 25.4 °C on average (range: 22.6–30.6 °C). The number of samples collected per tapstand is listed in Table S4.

**Table 1:** Water Quality Results at Baseline and Endline from Piped and Trucked Systems, at Point-of-Distribution (*T*_0_) and Point-of-Consumption (*T*_<1 h_ and *T*_3–24 h_)^a^

	piped water evaluation				trucked water evaluation			
	baseline	endline	difference	*p*-value	baseline	endline	difference	*p*-value
point-of-distribution (*T*_0_)	(*n* = 174)	(*n* = 212)			(*n* = 208)	(*n* = 213)		
*n* samples with FRC ≥ 0.2 mg/L (%)	87 (50%)	145 (68%)	+18%	<0.001	121 (58%)	181 (85%)	+27%	<0.001
FRC (mg/L)	0.20 (0.89, 0.01–3.84)	0.44 (0.80, 0.01–3.84)	+0.24 mg/L	<0.001	0.28 (0.46, 0.01–1.99)	0.92 (0.84, 0.01–3.48)	+0.64 mg/L	<0.001
TRC (mg/L)	0.35 (0.93, 0.01–3.89)	0.68 (0.88, 0.01–4.38)	+0.33 mg/L	<0.001	0.47 (0.55, 0.03–2.85)	1.12 (0.89, 0.01–3.90)	+0.65 mg/L	<0.001
air temperature (°C)	26.0 (2.2, 21.4–33)	24.4 (2.6, 19.8–36.7)	–2.4 °C	<0.001	27.9 (2.0, 21.9–35.0)	24.6 (2.6, 19.1, 32.3)	–3.3 °C	<0.001
water temperature (°C)	25.4 (1.5, 22.6–30.6)	24.1 (2.1, 20.0–34.3)	–1.3 °C	<0.001	25.5 (1.9, 20.0–31.1)	23.6 (1.7, 19.7–29.5)	–1.9 °C	<0.001
EC (μS/cm)	286 (62, 194–775)	296 (42, 63–588)		0.095	300 (21, 209–348)	313 (33, 2–372)	+13 μS/cm	<0.001
pH (unitless)	6.1 (0.58, 5.0–7.6)	6.1 (0.45, 5.0–7.0)		0.951	6.05 (0.56, 5.02–7.90)	5.72 (0.48, 5.09–6.99)	–0.33	<0.001
turbidity (NTU)	11.1 (9.2, 1.1–59.1)	11.2 (7.5, 0.1–56.5)		0.359	22.8 (21.9, 10.7–115)	17.5 (14.6, 0.1–60.5)	–5.3	<0.001
point-of-consumption (*T*_<1 h_)	(*n* = 174)	(*n* = 212)			(*n* = 208)	(*n* = 213)		
*n* samples with FRC ≥ 0.2 mg/L (%)	76 (44%)	132 (62%)	+18%	<0.001	109 (52%)	176 (83%)	+31%	<0.001
FRC (mg/L)	0.16 (0.83, 0.01–3.64)	0.39 (0.77, 0.01–4.23)	+0.23 mg/L	<0.001	0.22 (0.43, 0.01–1.83)	0.82 (0.85, 0.01–3.17)	+0.60 mg/L	<0.001
TRC (mg/L)	0.31 (0.88, 0.01–3.77)	0.55 (0.87, 0.01–4.41)	+0.24 mg/L	<0.001	0.36 (0.50, 0.02–2.30)	1.09 (0.89, 0.01–3.45)	+0.73 mg/L	<0.001
*E. coli* concentration (MPN/100 mL)	<1 (23, <1–100) (*n* = 19)				<1 (257, <1–1000) (*n* = 15)			
point-of-consumption (*T*_3–24 h_)	(*n* = 174)	(*n* = 212)			(*n* = 208)	(*n* = 213)		
follow-up time (h)	8 (7, 6–24)	19 (6, 6–24)			22 (5, 7–24)	22 (5, 6–24)		
*n* samples with FRC ≥ 0.2 mg/L (%)	37 (23%)	75 (35%)	+13%	0.003	16 (8%)	89 (42%)	+34%	<0.001
FRC (mg/L)	0.08 (0.5, 0.01–2.5)	0.11 (0.35, 0.01–1.84)	+0.03 mg/L	0.003	0.05 (0.14, 0.01–0.99)	0.17 (0.39, 0.01–1.99)	+0.12 mg/L	<0.001
TRC (mg/L)	0.20 (0.60, 0.01–3.39)	0.25 (0.40, 0.01–2.04)	+0.05 mg/L	0.043	0.14 (0.20, 0.03–1.19)	0.31 (0.45, 0.01–2.12)	+0.17 mg/L	<0.001
*E. coli* concentration (MPN/100 mL)	<1 (5, <1–14) (*n* = 19)				3.2 (334, <1–1000) (*n* = 16)			

a The results are presented as median (stdev, min–max), with *p*-values comparing baseline and endline results. Differences between baseline/endline are provided for *p* < 0.05.

**Figure 2:**
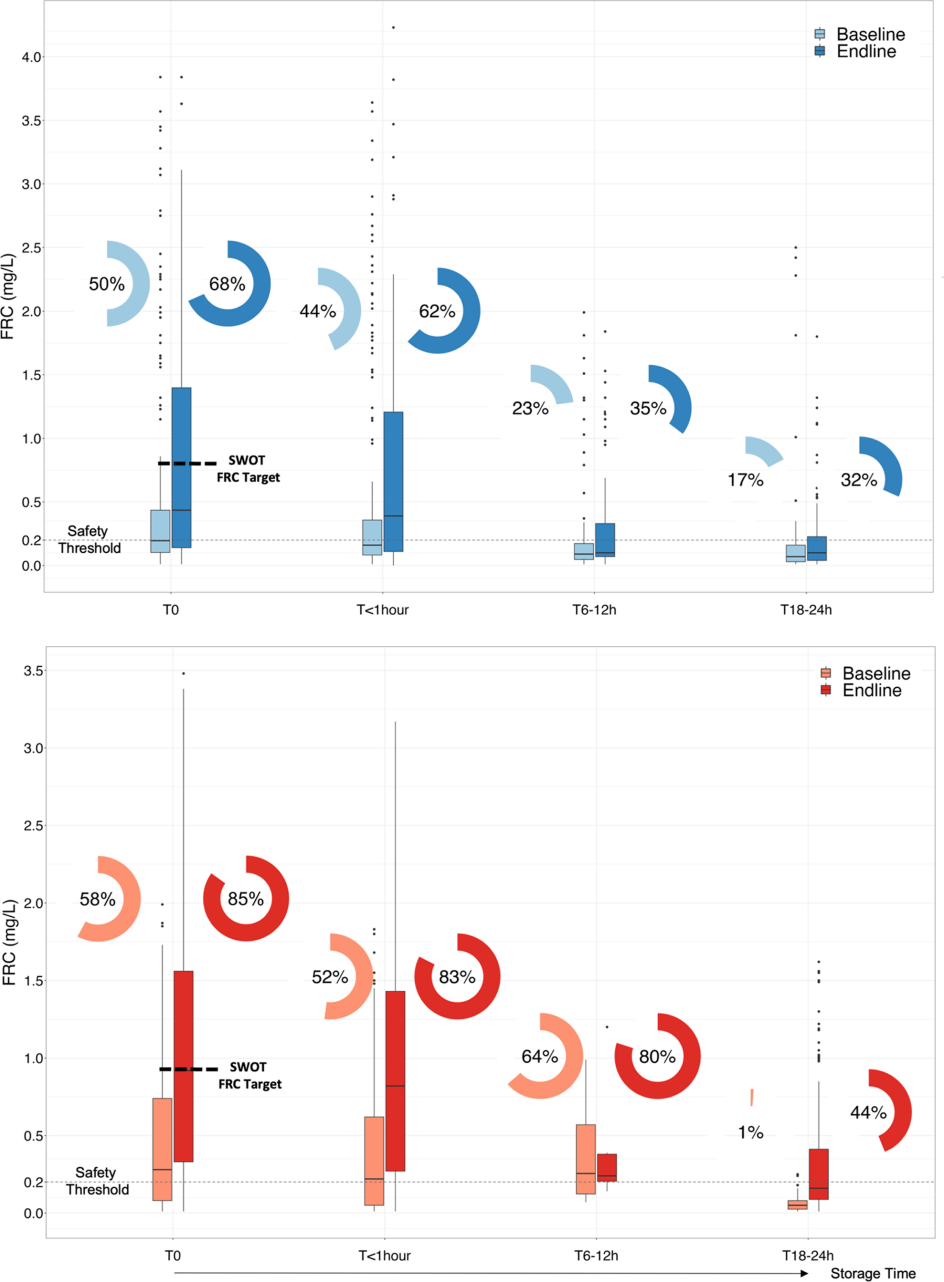
Point-of-distribution (*T*_0_) and point-of-consumption FRC results for the piped system (in blue) and trucked system (in red). The box-and-whisker bars are FRC (median and interquartile range), the circle pie charts are percent samples with FRC ≥ 0.2 mg/L, and *T*_0_ is the time of collection at point-of-distribution. *p*-values for differences between baseline and endline are significant (*p* ≤ 0.003) and are presented in Table 1.

The median initial visit point-of-consumption FRC was 0.16 mg/L, dropping to 0.08 mg/L at follow-up 3–24 h postcollection (Figure 2 and Table 1). Therefore, 77% of household samples had FRC < 0.2 mg/L. Please note that while 16% of baseline participants self-reported storing water for 18–24 h, 33% of water was sampled between 18–24 h, and key relevant time points are presented in Figure 2.

Thirty-eight samples were collected for microbiological analysis from 19 households. Point-of-consumption*E. coli* concentrations ranged <1–100 MPN/100 mL at the initial visit and <1–14 MPN/100 mL at 3–24 h (Table 1). One outlier sample with *E. coli* above the detection limit at point-of-distribution and within the detection range at point-of-consumption was dropped from the analysis (*n* = 37). Eleven samples (29%) did not meet the microbiological drinking water quality guideline value of <1 *E. coli* MPN/100 mL, one of which had ≥0.20 mg/L FRC at the point-of-consumption. Statistical analysis suggests ≥0.2 mg/L FRC was a good proxy for the absence of *E. coli* (*p* = 0.087 with Chi-square statistical test, *n* = 37).

At baseline, 55% (*n* = 119) of participants believed tapstand water was safe to drink and 20% (*n* = 43) because water smelled like chlorine (Table S1). From the chlorine T&O acceptability evaluation, the median population detection threshold was estimated at 0.56 mg/L, and the rejection threshold was 2.2 mg/L for the community served by the piped system.^17^

#### SWOT FRC Target

The SWOT generated a point-of-distribution FRC target of 0.7–0.8 mg/L to maintain FRC ≥ 0.2 mg/L for up to 12 h, the duration the majority of households self-reported storing water at baseline (68%, Table S1). The SWOT predicted this target would result in 65% of households having FRC ≥ 0.2 mg/L after 12 h of storage. Given FRC variability across the system (FRC ranged from 0.01 to 3.84 mg/L (Table 1)), four “sentinel” tapstands that were closest to the average FRC concentration for the system at baseline were selected for monitoring by Oxfam staff as the chlorine dosage was adjusted in each system tested.

#### Endline

Endline data were collected over 12 days during July–August 2022 from 223 households at all 17 tapstands sampled at the baseline. In total, 11 data points were removed; eight due to missing/mistimed data and three due to higher FRC at point-of-consumption than point-of-distribution. The final endline sample size was 212. Point-of-distribution FRC concentrations significantly increased to median 0.44 mg/L (Figure 2 and Table 1). These increases were not uniform as the median FRC at the four sentinel tapstands was 0.01, 0.28, 1.5, and 2.1 mg/L at sites 3, 4, 18, and 9, respectively. Thus, two sentinel tapstands did not reach the minimum SWOT target of 0.7–0.8 mg/L. pH and turbidity at point-of-distribution did not significantly change between baseline and endline (*p* = 0.951), while temperature decreased at endline (*p* < 0.001). Median initial point-of-consumption FRC was 0.39 mg/L, which dropped to 0.11 mg/L 3–24 h postcollection, with 65 samples (31%) stored for 6–12 h and 114 samples (54%) stored for 18–24 h (Table 1). The difference in median FRC concentrations between the point-of-consumption and 3–24 h postcollection was higher at endline (−0.28 mg/L) than at baseline (−0.08 mg/L).

Overall, 35% of household point-of-consumption samples had FRC ≥ 0.2 mg/L for water stored up to 24 h, which was a significant increase from baseline (+13%, *p* = 0.003) (Figure 2 and Table 1). This increase was observed despite an increase in samples collected after longer storage times at the endline, which could have skewed data. Overall, the partial implementation of the SWOT FRC target recommendation improved piped system point-of-consumption FRC but did not achieve the theoretical point-of-consumption improvements possible with full implementation. Wilcoxon rank-sum tests indicated point-of-consumption FRC was associated with spatial variability (tapstand (*p* < 0.001) and zone (*p* < 0.001)), highlighting the importance of spatial variability in water distribution system analysis.

More participants at endline had received messages about chlorinated water (20% compared to 13%, *p* < 0.001), thought they could get sick from water (90% compared to 79%, *p* = 0.060), and believed tapstand water was safe to drink (79% compared to 55%, *p* < 0.001) (Table S1). The number of participants who reported detecting changes in their water in the weeks before the survey was similar at endline and baseline (65 and 64%, respectively), suggesting that the increase in point-of-distribution FRC at endline may not have been noticed by users. Additionally, the number of participants reporting tapstand water had a good taste and smell at endline doubled (to 56 and 67% for taste and smell, respectively, at endline, compared to 25 and 34% at baseline, *p* < 0.001). This suggests a positive perception of chlorine taste and odor among endline participants, in line with findings regarding the number of participants who liked having chlorine in drinking water because it smells clean (increase from 9 to 17% from baseline to endline, *p* = 0.017), which is common in emergency contexts where populations perceive risk as high.^2^

#### DBP Concentrations at Endline

Median combined THMs concentrations were 97 μg/L (range 9–146, *n* = 20) and 105 μg/L (range 35–152, *n* = 20) at point-of-distribution and point-of-consumption, respectively, using the Hach THM Plus field method. Median summed THMs concentrations were 77 μg/L (range 6–100, *n* = 20) and 79 μg/L (range 8–96, *n* = 20) at point-of-distribution and point-of-consumption, respectively, using the USEPA standard method. Overall, combined THMs concentrations (from either method) did not exceed either the chloroform individual analyte WHO guideline value of 300 ppb or the summed individual guideline values of 560 ppb.^3^ Additionally, concentrations were not significantly different between point-of-distribution and point-of-consumption for either method (*p* = 0.213 field method; *p* = 0.507 laboratory method). This suggests a low influence of storage duration on THMs concentration. These methods led to significantly different combined or summed THMs results (*p* < 0.001). Higher concentrations were recorded by the field method, potentially because of including other interfering compounds, and/or because the laboratory method underestimated THMs because samples were not acidified to pH < 2 before shipment to the laboratory, and the holding time for the USEPA method 8260C was exceeded by 2 days.

The median FRC concentrations were 0.24 mg/L at the point-of-distribution and 0.15 mg/L at the point-of-consumption in samples selected for THMs analysis. The combined THMs concentration for the highest FRC concentration in the system (if the recommended SWOT FRC target of 0.80 mg/L was reached) was estimated at 267 and 133 μg/L using field and laboratory methods, respectively (upper 95th percentile, quantile regression modeling). Therefore, modeling indicated reaching the upper range of the recommended SWOT FRC target would not result in combined THMs concentrations exceeding international guideline values.

### Trucked System Evaluation

#### Site and Population

Two distribution tanks of 10 m^3^ each were included, with 224 and 225 participants recruited at baseline and endline, respectively, on days distribution tanks were filled. Full details of the population are presented in Table S2. Please note that the trucked system evaluation is succinctly presented due to space limitation.

#### Baseline

Data were collected over 10 days in June 2022. Of 224 paired samples, 16 were removed (11 due to missing/mistimed data, five due to higher FRC at point-of-consumption than point-of-delivery), for a final sample size of 208. At baseline, 54% of participants reported storing water <12 h, 29% 12–18 h, and none for >24 h (Table S2). At the point-of-distribution, 58% of samples had FRC ≥ 0.20 mg/L (median 0.28 mg/L) (Table 1). Water pH was within the range of 5.0–7.9 for effective chlorination, but median turbidity exceeded recommendations at 22.8 NTU (range 10.7–115).

Initial median point-of-consumption FRC was 0.22 mg/L (range 0.01–1.83), dropping to 0.05 mg/L (range 0.01–0.99) after 3–24 h storage (Table 1). Overall, 52% of point-of-consumption samples had FRC > 0.2 mg/L initially, compared to 8% after 3–24 h of storage. For microbiological analysis, 31 samples from 18 households were collected. Ten samples (33%) did not meet the microbiological drinking water quality standard (<1*E. coli* MPN/100 mL), and statistical analysis did not confirm a relationship between FRC ≥ 0.20 mg/L and <1*E. coli* in the trucked system (*p* = 0.205, *n* = 30).

In surveys, 62% of participants believed water at distribution tanks was safe to drink and 28% reported receiving messaging about chlorinated water (Table S2). About half of the participants thought water from distribution tanks had a good taste (51%) and smell (45%). The estimated median population chlorine detection and rejection thresholds were 1.4 and 1.8 mg/L, respectively.^17^

#### SWOT FRC Targets

The SWOT generated a point-of-distribution FRC target for the trucked system of 0.9 mg/L to protect water for up to 12 h postdistribution, to strike a balance between achieving FRC targets and ensuring people would not reject chlorinated water.

#### Endline

Endline data was collected over 9 days in August 2022. Of 225 paired samples, 12 were removed (six due to missing/mistimed data, six due to higher FRC at point-of-consumption than point-of-delivery), for a final sample size of 213. Point-of-distribution FRC concentrations significantly increased to median 0.92 mg/L (range 0.01–3.48), indicating that the SWOT FRC target recommendation was successfully achieved (Table 1). We observed high FRC variability between the two points of distribution, between truck deliveries, and over time, which highlights the challenge of achieving consistent FRC concentrations across batches. In addition, other water quality parameters significantly changed between baseline and endline, with decreasing turbidity, pH, and temperature and increasing electrical conductivity (*p* < 0.001 for all parameters). These changes may be due to logistical constraints leading Oxfam to change from a 20 m^3^ truck at baseline to an 8 m^3^ truck at endline.

Point-of-consumption storage times remained similar between baseline and endline (median 22 h) (Table 1). The proportion of households with FRC ≥ 0.2 mg/L at point-of-consumption significantly increased to 42% at endline (+34% improvement from baseline) (*p* < 0.001). More participants at endline (74%) believed the water they collected was safe to drink (62% at baseline; *p* = 0.017). The number of participants who reported detecting any change in taste or smell significantly increased at endline (*p* < 0.001), in line with the achievement of the higher target FRC recommended by the SWOT.

### Experience of Water System Operators

At the baseline, six interviews were conducted with Oxfam water system operators. A main challenge discussed was the complexity of using manual batch chlorination to meet point-of-distribution FRC targets. Informants noted the need to work quickly when chlorinating water to deliver water in a timely manner, potentially limiting contact times. Informants also discussed the challenges of overnight water storage and chlorine decay in a large, piped network. These factors contributed to variable FRC concentrations throughout the system and made it difficult for operators to link tapstand FRC monitoring to a specific chlorinated water batch. Informants also discussed resource constraints that impacted water chlorination, including running out of water treatment chemicals (e.g., alum, lime), having no scale to weigh HTH powder, no digital FRC tester to provide accurate measurements, a lack of human resources for ongoing water quality monitoring, and a high turnover among the operator team. Altogether, these factors made it difficult to build institutional memory and improve water chlorination practices. One informant said that “[monitoring] is one of the weaknesses that we have always had because data collection and also [...] analysis is done in a rudimentary manner.” Because of these challenges, some informants discussed frequent errors in dosing chlorine and maintaining FRC targets. Lastly, informants discussed that feedback from the community was meant to inform practices (but this was seldom done) and that operators received mixed community feedback, which made it difficult to adjust chlorination practices.

Three interviews were conducted with the same water system operators 2–3 weeks after endline data collection concluded. Informants stated that SWOT implementation had helped them to improve chlorination operations by adjusting dosage but also by being more mindful of contact time. One informant said that “When the SWOT came in, they [the operators] realized maybe some steps were being missed and some work was always not being done as expected.” They felt the operator capacity had increased, and they had more knowledge about water chlorination and water quality monitoring. One informant said that they were now “looking at everything cautiously, reasoning out everything, doing more follow-ups with the team”. According to informants, SWOT and this study provided guidance on what a good monitoring plan was, including how to incorporate data collection at the household level, and that it helped them collect data in a more organized manner. However, one informant mentioned that data recording had not changed and that they were uncomfortable using the SWOT independently to generate a target. They recommended conducting more trainings, including on data collection and management, and with “hands-on” exercises. The main recommendations provided by endline informants were to have the technology and equipment to avoid human errors during dosage; additional human and equipment resources to conduct proper water quality monitoring; and to address challenges related to the large network size.

## Discussion

We conducted two before-and-after mixed-methods evaluations in the Kyaka II settlement to evaluate the SWOT’s effectiveness with surface water supplies in humanitarian settings. We observed that point-of-distribution water quality did not reliably meet standards at baseline; SWOT-generated FRC targets increased tapstand FRC concentrations and did not exceed taste and odor thresholds; SWOT-generated target FRCs produced water below guideline values for DBPs; and maximal SWOT effectiveness was not achieved due to operational challenges implementing SWOT FRC targets in these surface water systems.

At baseline, water quality at point-of-distribution in both piped and trucked systems did not reliably meet minimum standards. In addition to having turbidity > 5 NTU, 42–52% of point-of-distribution water samples had FRC < 0.2 mg/L. These low point-of-distribution FRC concentrations led to low household point-of-consumption FRC. Additionally, more than one-quarter of participants reported they believed tapstand water was not safe to drink, and some households reported not always drinking tapstand water because of lack of water and/or crowding. These results highlight the need for improved chlorination programs at Kyaka II.

In both piped and trucked systems, SWOT-generated chlorination targets recommended increasing point-of-distribution FRC concentrations. After implementation of SWOT targets, the proportion of households with point-of-consumption FRC ≥ 0.2 mg/L up to 24 h after distribution increased by 13 and 34% in piped and trucked systems, respectively. SWOT FRC targets were also within observed chlorine taste and odor acceptability thresholds for both populations, and we did not observe an increase in the rejection of water with increased chlorination targets. Although participants noticed a change in their water in the weeks following SWOT target implementation in the trucked water evaluation, the number of participants reporting that water had good taste and smell at endline increased or stayed similar, and more participants believed point-of-distribution water was safe to drink at endline. We found SWOT target recommendations helped optimize point-of-consumption FRC while balancing user preferences. Lastly, we found limited, nonstatistical corroborating evidence that having ≥0.2 mg/L FRC indicates low risk for *E. coli* presence and is a meaningful indicator of microbiological water safety.

In this study, DBPs concentrations remained below WHO guidelines, even after increasing point-of-distribution FRC concentrations. Worst-case scenarios from quantile regression modeling indicated DBPs concentrations would not exceed WHO guideline values even if FRC concentrations at tapstands were elevated to SWOT-recommended targets. These results corroborate the findings of previous studies^10−12^ showing DBPs are below WHO guideline values in chlorinated water supplies in humanitarian settings.

While point-of-consumption FRC concentrations did increase in our study, the expected proportion of households with FRC ≥ 0.2 mg/L associated with implementing the SWOT targets was not achieved in either system at Kyaka II, and gains were smaller than those achieved in previous studies.^5,6^ We observed that effective implementation of SWOT FRC targets at Kyaka II was conditioned by operational challenges with dosing and control of chlorination processes, which led to variability in point-of-distribution FRC concentrations. Surface waters, often with high turbidity and other contaminants, are more challenging to chlorinate, and prechlorination clarification is recommended.^1^ These operational challenges explain why we observed greater FRC improvements in the trucked rather than the piped system because SWOT FRC targets were able to be more effectively implemented in the smaller, less complex trucked system where operators could more easily control FRC concentrations. This was in contrast to the piped system, which was extensive (>17 km of distribution lines) and more complex (balancing FRC across the network, water stagnating in pipes between batches, etc.) to chlorinate. These results suggest that the most effective way to implement the SWOT is to generate FRC targets for the smallest unit of chlorination control possible. When the SWOT is implemented in larger piped networks, there is a need to integrate spatial variability and distribution system decay modeling into SWOT modeling. Additionally, the SWOT should be regularly updated with latest monitoring data to account for differences in water quality (e.g., turbidity, temperature) and chlorine decay that arise due to seasonal changes in weather and environmental conditions.^6^

Despite the fact that the surface water source and treatment were the same for the piped and trucked water evaluations, there were considerable ranges of water quality results both within and between the piped and trucked water evaluations. These variabilities could be due to differences in the day-to-day effectiveness of the treatment processes in a humanitarian context; the season of data collection; and potential contamination in the long piped system (compared to a single truck) during delivery. These differences all influence chlorine demand, and as such, the piped and trucked water evaluations are not directly comparable.

These findings indicate that to fully optimize point-of-consumption FRC in surface water supplies in humanitarian settings, the SWOT must extend its support beyond just generating site-specific chlorination targets to also providing broad-based technical support to water system operators. This includes equipment (e.g., chlorometers, dosing scales, digital survey tools) and training support on water treatment processes (e.g., clarification and chlorination), chlorine dosage management, water quality monitoring, and protecting treated water during distribution. Such investments would support the delivery of more consistent quality water and better ensure the desired public health goal of safe water supply is achieved. However, while capacity building and technical assistance to operators are necessary, they are insufficient to optimize FRC levels without the SWOT. Well-capacitated operators might stabilize FRC, but not to a site-specific optimized FRC target. Lastly, there are costs associated with SWOT implementation. A recent review found the SWOT added costs because of the need to test FRC at point-of-consumption in addition to point-of-distribution.^19^ In programs already testing FRC at point-of-consumption, as recommended in humanitarian sector guidelines,^1^ the added cost for the SWOT was relatively low.

Limitations of this work include low adherence to recommended SWOT FRC targets, which reduced the opportunity to fully assess SWOT effectiveness in improving point-of-consumption FRC. Additionally, Oxfam used the SWOT with the support of the SWOT team, which limits the evidence on response organization personnel independently implementing the SWOT. THM samples for the laboratory method were not acidified and exceeded their hold time, which could have resulted in an underestimation of laboratory THMs concentrations. While 888 paired samples were attempted to be collected, 29 (3.3%) were dropped due to FRC being higher at point-of-consumption than point-of-delivery, potentially because household inaccurately reported water practices such as water treatment. Relatedly, we worked to ensure that the same water was sampled at initial and follow-up visits, but there could have been inaccuracies in reporting that affected this testing. Lastly, data from the piped and trucked systems were not compared, as although all water originated at the Sweswe water treatment plant, the evaluations were conducted in different months with different water qualities and chlorine demands. Future research is needed to determine how to operationalize the training needed for optimal SWOT implementation.

Overall, we found that the SWOT can improve point-of-consumption FRC concentrations in humanitarian water systems using surface water sources without increasing chlorine taste and odor rejection or exceeding WHO THMs guidelines values. However, SWOT FRC targets were only partially achieved due to operational challenges and spatial variability in the large piped system. Therefore, we recommend that the SWOT incorporate technical support for water system operators on broader water treatment and monitoring topics and incorporate spatial variability into modeling. These steps will help improve operational implementation of SWOT FRC targets and enhance SWOT effectiveness for improving water quality and, ultimately, reducing public health risks.

## Data Availability

The data underlying this study are openly available in Tufts University Box at https://tufts.box.com/s/3b5ue5xg2gnj5hcli01cgl50ipt7x8ka.

## Supporting Information Available

Map of the evaluated piped water system, respondents’ and data collection characteristics, and the initial and follow-up surveys (PDF)

## References

[ref1] Sphere Handbook: Humanitarian Charter and Minimum Standards in Humanitarian Response, 4th ed.; Sphere Association: Geneva, Switzeland, 2018.

[ref2] Branz A.; Levine M.; Lehmann L.; Bastable A.; Ali S.; Kardir K.; Yates T.; Bloom D.; Lantagne D. Chlorination of Drinking Water in Emergencies: A Review of Knowledge to Develop Recommendations for Implementation and Research Needed. Waterlines 2017, 36 (1), 4–39. 10.3362/1756-3488.2017.002.

[ref3] Guidelines for Drinking-Water Quality, 4th ed.; World Health Organization: Geneva, Switzerland, 2017.

[ref4] Ali S. I.; Ali S. S.; Fesselet J.-F. Effectiveness of Emergency Water Treatment Practices in Refugee Camps in South Sudan. Bull. World Health Organ. 2015, 93 (8), 550–558. 10.2471/BLT.14.147645. 26478612 PMC4581656

[ref5] Ali S. I.; Jean-François F.; Arnold M.; Khan U.; Ali S. S.; Spendlove M.; Vasileiou A.; Santi M. D.; Rahma S.; Adler D.; Orbinski J. Development of a Machine-Learning Enabled Safe Water Optimization Tool for Humanitarian Response. F1000Research 2020, 1, e1 10.7490/F1000RESEARCH.1117910.1.

[ref6] Ali S. I.; Ali S. S.; Fesselet J.-F. Evidence-Based Chlorination Targets for Household Water Safety in Humanitarian Settings: Recommendations from a Multi-Site Study in Refugee Camps in South Sudan, Jordan, and Rwanda. Water Res. 2021, 189, 116642 10.1016/j.watres.2020.116642. 33246215

[ref7] Carmo R. F.; Bevilacqua P. D.; Barletto M. Social Representations of Drinking Water: Subsidies for Water Quality Surveillance Programmes. J. Water Health 2015, 13 (3), 671–679. 10.2166/wh.2015.171. 26322753

[ref8] Mitro B.; Wolfe M. K.; Galeano M.; Sikder M.; Gallandat K.; Lantagne D. Barriers and Facilitators to Chlorine Tablet Distribution and Use in Emergencies: A Qualitative Assessment. Water 2019, 11 (6), 1121 10.3390/w11061121.

[ref9] Lantagne D.; Clasen T. Point-of-Use Water Treatment in Emergency Response. Waterlines 2012, 31 (1–2), 30–52. 10.3362/1756-3488.2012.005.

[ref10] Lantagne D. S.; Blount B. C.; Cardinali F.; Quick R. Disinfection By-Product Formation and Mitigation Strategies in Point-of-Use Chlorination of Turbid and Non-Turbid Waters in Western Kenya. J. Water Health 2008, 6 (1), 67–82. 10.2166/wh.2007.013. 17998608

[ref11] Cardinali F.; Lantagne D. S.; Blount B. C. Disinfection By-Product Formation and Mitigation Strategies in Point-of-Use Chlorination with Sodium Dichloroisocyanurate in Tanzania. Am. J. Trop. Med. Hyg. 2010, 83 (1), 135–143. 10.4269/ajtmh.2010.09-0431. 20595492 PMC2912590

[ref12] Ali S. I.; Arnold M.; Liesner F.; Fesselet J.-F. Characterization of Disinfection By-Products Levels at an Emergency Surface Water Treatment Plant in a Refugee Settlement in Northern Uganda. Water 2019, 11 (4), 647 10.3390/w11040647.

[ref13] De Santi M.; Khan U. T.; Arnold M.; Fesselet J.-F.; Ali S. I. Forecasting Point-of-Consumption Chlorine Residual in Refugee Settlements Using Ensembles of Artificial Neural Networks. npj Clean Water 2021, 4 (1), 35 10.1038/s41545-021-00125-2.

[ref14] Standard Practice for Determination of Odor and Taste Thresholds By a Forced-Choice Ascending Concentration Series Method of Limits (E679-19) ASTM (American Society for Testing and Materials): 2019.

[ref15] Standard Methods For the Examination of Water and Wastewater American Public Health Association; American Water Works Association; Water Environment Federation: 2010.

[ref16] Osler A. L.; Alfredo K. A.; Mihelcic J. R. Chlorine Water Taste Threshold and Acceptability among Indigenous and Non-Indigenous Populations in Rural Panama. Environ. Sci. Technol. 2024, 58 (12), 5548–5556. 10.1021/acs.est.3c05630. 38471095

[ref17] Heylen C.; String G.; Ali S. I.; Brown J.; Fesselet J.-F.; Lantagne D.; Naliyongo D.; Ogira V.; Orbinski J.; De Santi M. Evaluating Chlorine Taste and Odor Acceptability to Inform Drinking Water Chlorination in Humanitarian Settings: Lessons from Trials in Uganda. PLOS Water October 20th (PWAT-D-24-00060).

[ref18] How to Determine Dosage for Batch Chlorination (Modified Horrocks’ Method) Centre for Affordable Water and Sanitation Technology: Alberta, Canada; 2021.

[ref19] Komuhangi C.; Knight J. Humanitarian Innovation Fund Case Study: Safe Water Optimization Tool 2023 https://www.elrha.org/wp-content/uploads/2023/11/Elrha-HIF-Case-Study-SWOT.pdf.

