# Supplementary material for: Evaluation of the Safe Water Optimization Tool to Provide Evidence-Based Chlorination Targets in Surface Waters: Lessons from a Refugee Setting in Uganda
Source: Environ Sci Technol. 2024 Oct 9;58(42):18531–40. doi: 10.1021/acs.est.4c04240 (PMC11500394; doi:10.1021/acs.est.4c04240)
Supplement: Supplementary file 1 — es4c04240_si_001.pdf [file es4c04240_si_001.pdf]

Supporting Information

Evaluation of the Safe Water Optimization Tool to provide evidence-based chlorination targets in surface waters: lessons from a refugee setting in Uganda

Camille Heylen[1*], Gabrielle String[1,2], Doreen Naliyongo[3], Syed Imran Ali[4], James Brown[4], Michael De Santi[4], Vincent Ogira[4], Jean-François Fesselet[5], James Orbinski[4], and Daniele Lantagne[1,6]

1: Tufts University, Civil and Environmental Engineering, Medford, MA, 02155, United States

2: Lehigh University, Civil and Environmental Engineering, Bethlehem, PA, 18015, United States

3: Oxfam, Kampala, P.O. Box 6228, Uganda

4: York University, Dahdaleh Institute for Global Health Research, Toronto, ON M3J 2S5, Canada

5: Médecins Sans Frontières, Amsterdam, 1018 DD, The Netherlands

6: Tufts University, Friedman School of Nutrition, Boston, MA, 02111, United States

Summary: 20 pages - 1 figure, 4 tables, and 2 survey templates.

Figure S1: Map of Piped Water Network from Nyaburungi Reservoir showing tapstands where baseline and endline water quality monitoring was conducted for the piped surface water system evaluation. Tapstands 3, 4, 9, and 18 were selected as sentinel sites to monitor the SWOT FRC target implementation because they were closest to the average FRC measurements for the system at baseline. S3

Table S1: Participants' characteristics for the baseline and endline data collection of the piped surface water system evaluation. P-values from statistical analysis compare whether or not there were statistically significant differences (p<0.05) in the baseline and endline populations' characteristics...... S4

Table S2: Participants' characteristics in baseline and endline data collection in the trucked surface water system evaluation. P-values indicate whether or not differences were statistically significant differences (p<0.05) in the baseline and endline populations' characteristics. S6

Table S3: Summary of the number of samples collected during the study (n=2,768). S8

Table S4: Breakdown of FRC samples collected at the point of distribution at baseline and endline and included in the analysis, by distance from the treatment plan. S8

Annex S1: Water Quality Testing and Survey: Initial Survey (Control and Intervention Groups)..........S9

Annex S2: Water Quality Testing and Survey: Follow-up Survey (Control and Intervention Groups).. S18

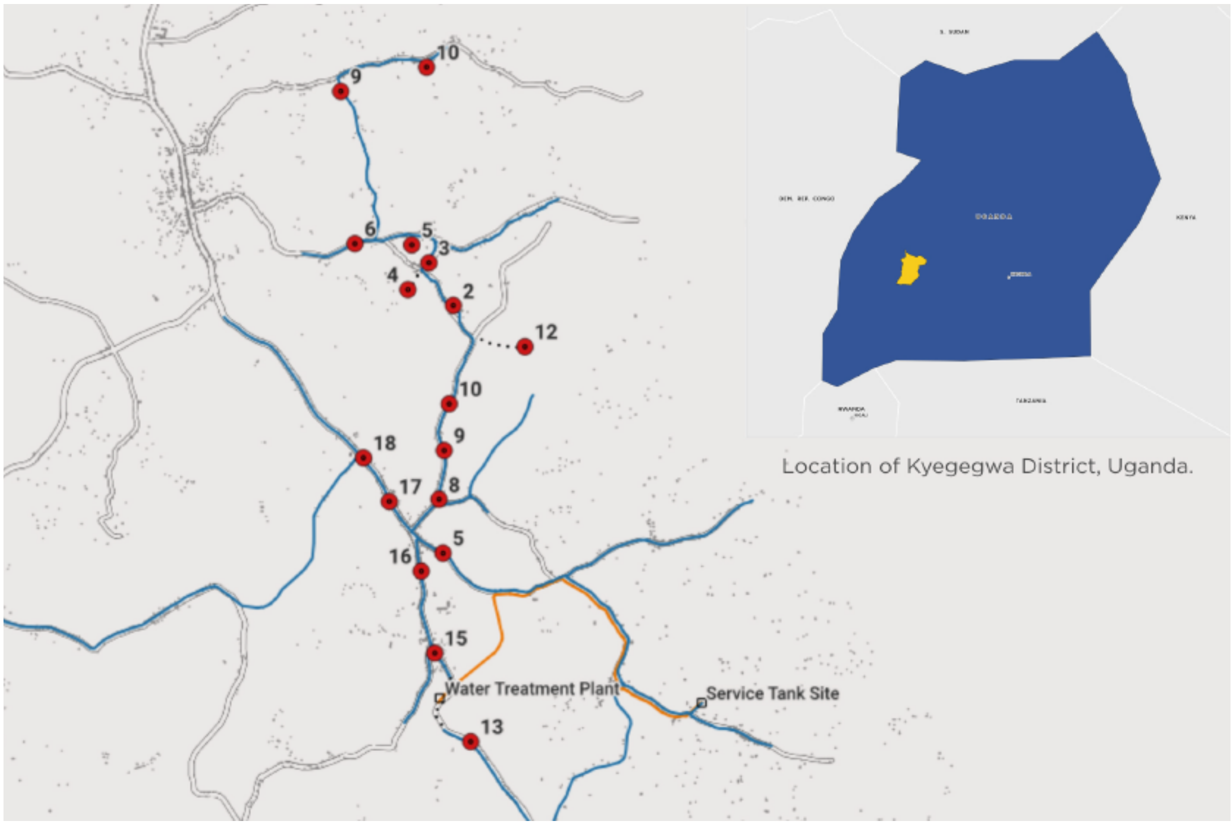

Figure S1: Map of Piped Water Network from Nyaburungi Reservoir showing tapstands where baseline and endline water quality monitoring was conducted for the piped surface water system evaluation. Tapstands 3, 4, 9, and 18 were selected as sentinel sites to monitor the SWOT FRC target implementation because they were closest to the average FRC measurements for the system at baseline.

Table S1: Participants' characteristics for the baseline and endline data collection of the piped surface water system evaluation. P-values from statistical analysis compare whether or not there were statistically significant differences ($p<0.05$) in the baseline and endline populations' characteristics.

	**Baseline (N=216)**	**Endline (N=223)**	**p-value**
**Socio-demographic characteristics**			
N Male (%)	36 (17)	55 (25)	0.051
Median age (SD, min-max)	32 (11, 18-74)	31 (8, 19-62)	0.78
Median number of people living in participant's home (SD, min-max)	5 (2.4, 1-14)	5 (2, 1-13)	0.797
N Participants went to school (%)	121 (56)	116 (52)	0.456
N Female head of house is able to read and write (%)	90 (42)	89 (40)	0.355
N Male head of house is able to read and write (%)	99 (46)	94 (42)	0.166
N Participants living in the area for more than 1 year (%)	190 (88)	199 (89)	0.787
**WASH situation**			
N Participants who don't have a dedicated space to wash their hands in home (%)	198 (92)	200 (90)	0.391
N Participants who have a private latrine (%)	154 (71)	169 (76)	0.268
N Participants with distance between the latrine and the point-of-consumption <5 m (%)	74 (35)	40 (19)	**<0.001**
**Transport containers characteristics**			
N Participants with jerrycan (%)	215 (100)	222 (>99)	>0.999
N Participants with 20L container (%)	190 (88)	161 (72)	**<0.001**
N Participants with small-opening container (%)	211 (99)	221 (>99)	0.963
N Participants with one container (%)	73 (34)	182 (82)	**<0.001**
N Participants with covered container (%)	177 (83)	206 (92)	**0.003**
N Participants with dirty* container (%)	124 (58)	142 (64)	0.259
N Participants who clean the transport container (%)			
on a daily basis	84 (39)	63 (28)	
on a weekly basis	112 (52)	127 (57)	0.098
rarely	15 (7)	22 (10)	
don't clean the transport container	5 (2)	10 (4)	
N Participants who cleaned transport container with (%)			
water from the tapstand	152 (70)	200 (90)	**<0.001**
soap	157 (73)	139 (63)	**0.032**
rocks/sand	152 (70)	164 (74)	0.477
bleach	29 (13)	16 (7)	**0.047**
other	30 (14)	6 (3)	**<0.001**
N Participants who obtained the transport container more than 1 year ago (%)	148 (68)	129 (58)	0.068
N Participants who stored the transport container inside when empty (%)	206 (95)	216 (97)	0.574
**Collection practices**			
N Participants who collect their water from the tapstand in the morning (%)	212 (98)	214 (96)	0.286
N Participants who collect their water from the tapstand in the afternoon (%)	56 (26)	120 (54)	**<0.001**
N Participants who collect their water from the tapstand in the evening (%)	136 (63)	146 (65)	0.654
N Participants who always collect water from a tapstand (%)	182 (84)	212 (95)	**<0.001**
N Participants who don't always collect water from the same tapstand because (%)			
it is too crowded	30 (14)	25 (11)	0.482
no water is running	197 (91)	209 (94)	0.413
too far	0	3 (1)	0.258
**Storage practices**			
N Participants storing collected water for (%)			
N < 3 hours	17 (8)	2 (1)	
N 3-6 hours	66 (31)	13 (6)	
N 6-12 hours	62 (29)	113 (51)	**<0.001**
N 12-18 hours	22 (10)	57 (26)	
N 18-24 hours	34 (16)	37 (17)	
N >24 hours	15 (7)	0	
N Participants who transferred water from transport to storage container (%)	23 (11)	11 (5)	**0.039**

**Messaging about chlorinated water**			
N Participants who have received any messaging about the chlorinated water (%)	28 (13)	44 (20)	**<0.001**
N Participants who like about having chlorine in drinking water that (%)			
it makes safe	129 (60)	146 (65)	0.252
it has a good taste	20 (9)	28 (12)	0.340
it smells clean	20 (9)	39 (17)	**0.017**
it makes people less sick	77 (36)	51 (23)	**0.005**
N Participants who dislike about having chlorine in drinking water because (%)			
chemicals are bad	29 (13)	54 (24)	**0.006**
it has a bad taste	67 (31)	63 (28)	0.596
it has a bad smell	100 (46)	61 (27)	**<0.001**
it makes the stomach hurt	11 (5)	19 (8)	**0.217**
it makes cough	22 (10)	40 (18)	**0.028**
it makes the skin itch	39 (18)	35 (17)	0.594
**Attitudes**			
N Participants who think they or any member of their family can get sick from water (%)	171 (79)	201 (90)	**0.006**
N Participants who think they can get diarrhea from drinking water (%)	93 (46)	155 (72)	**<0.001**
N Participants who know water is safe to drink because (%)			
water is clear	191 (88)	214 (96)	**0.006**
it comes from the point-of-distribution	28 (13)	25 (11)	0.677
water is treated	82 (38)	69 (31)	0.148
it has chlorine smell or odor	49 (23)	33 (15)	**0.046**
N Participants who know water is not safe to drink because (%)			
it looks dirty	200 (93)	217 (97)	**0.041**
it makes sick	50 (23)	88 (39)	**<0.001**
it smells bad	74 (66)	121 (54)	**<0.001**
it tastes bad	46 (21)	90 (40)	**<0.001**
N Participants who believe the water they have collected today at the tapstand is safe to drink (%)	119 (55)	177 (79)	**<0.001**
N Participants who believe the water they have collected today at the tapstand is safe to drink because it has chlorine smell or odor (%)	43 (20)	25 (11)	**0.017**
N Participants who think the water collected at the tapstand has a good taste (%)	55 (25)	124 (56)	**<0.001**
N Participants who think the water collected at the tapstand has a good smell (%)	73 (34)	150 (67)	**<0.001**
N Participants who have detected any particular change in their water these past few weeks (%)	139 (64)	144 (65)	**0.120**

**Dirty* being defined as being if the container has any visible dirt or green algae growing on the inside of the container.

Table S2: Participants' characteristics in baseline and endline data collection in the trucked surface water system evaluation. P-values indicate whether or not differences were statistically significant differences (p<0.05) in the baseline and endline populations' characteristics.

	Baseline (N=224)	Endline (N=225)	p-value
**Socio-demographics**			
N Male (%)	45 (20)	61 (27)	0.113
Median age (SD, min-max)	30 (9, 18-72)	28 (6, 18-63)	**<0.001**
Median number of people living in participant's home (SD, min-max)	5 (1.7, 1-12)	6 (2.1, 1-12)	**0.001**
N Participants went to school (%)	67 (30)	101 (45)	**0.001**
N Female head of house is able to read and write (%)	51 (23)	75 (33)	**<0.001**
N Male head of house is able to read and write (%)	74 (33)	87 (39)	**0.025**
N Participants living in the area for more than 1 year (%)	202 (89)	205 (91)	0.811
**WASH situation**			
N Participants who don't have a dedicated space to wash their hands in homes (%)	209 (93)	207 (92)	0.095
N Participants who have a private latrine (%)	159 (71)	150 (67)	0.060
N Participants with distance between the latrine and the point-of-consumption is <5 m (%)	73 (34)	90 (42)	**<0.001**
**Transport containers characteristics**			
N Participants with jerrycan (%)	216 (96)	217 (97)	0.612
N Participants with 20L container (%)	92 (41)	120 (54)	**<0.001**
N Participants with small-opening container (%)	217 (96)	215 (96)	0.992
N Participants with one container (%)	207 (92)	115 (51)	**<0.001**
N Participants with covered container (%)	45 (20)	56 (25)	0.248
N Participants with dirty* container (%)	140 (62)	175 (78)	**<0.001**
N Participants who clean the transport container (%)			
on a daily basis	71 (32)	74 (33)	
on a weekly basis	82 (36)	99 (44)	0.008
rarely	49 (22)	45 (20)	
don't clean the transport container	23 (10)	6 (3)	
N Participants who cleaned transport container with (%)			
water from the tapstand	154 (68)	203 (91)	**<0.001**
soap	137 (61)	116 (52)	0.065
rocks/sand	150 (67)	159 (71)	0.376
bleach	5 (2)	45 (20)	**<0.001**
other	1 (<1)	2 (<1)	0.997
N Participants who obtained the transport container more than 1 year ago (%)	152 (68)	137 (61)	0.217
N Participants who stored the transport container inside when empty (%)	225 (>99)	175 (78)	**<0.001**
**Collection practices**			
N Participants who collect their water from the tapstand in the morning (%)	207 (92)	202 (90)	0.608
N Participants who collect their water from the tapstand in the afternoon (%)	142 (63)	142 (63)	1
N Participants who collect their water from the tapstand in the evening (%)	154 (68)	162 (72)	0.426
N Participants who always collect water from a tapstand (%)	163 (73)	224 (>99)	**<0.001**
N Participants who don't always collect water from the same tapstand because (%)			
it is too crowded	58 (26)	1 (<1)	**<0.001**
no water is running	214 (95)	220 (98)	0.117
too far	27 (12)	3 (1)	**<0.001**
**Storage practices**			
N Participants storing collected water for (%)			
N < 3 hours	9 (4)	0	
N 3-6 hours	38 (17)	12 (5)	
N 6-12 hours	74 (33)	137 (61)	**<0.001**
N 12-18 hours	65 (29)	51 (23)	
N 18-24 hours	36 (16)	24 (11)	
N >24 hours	2 (<1)	0	

N Participants who transferred water from transport to storage container (%)	21 (9)	4 (2)	**<0.001**
**Messaging about chlorinated water**			
N Participants who have received any messaging about the chlorinated water (%)	62 (28)	30 (13)	**<0.001**
N Participants who like about having chlorine in drinking water that (%)			
it makes safe	125 (56)	75 (34)	**<0.001**
it has a good taste	23 (10)	54 (24)	**<0.001**
it smells clean	22 (10)	86 (39)	**<0.001**
it makes people less sick	51 (23)	52 (23)	0.959
N Participants who dislike about having chlorine in drinking water because (%)			
chemicals are bad	56 (25)	60 (27)	0.704
it has a bad taste	60 (27)	42 (19)	0.062
it has a bad smell	55 (24)	30 (13)	**0.004**
it makes the stomach hurt	48 (21)	12 (5)	**<0.001**
it makes cough	26 (12)	16 (7)	0.153
it makes the skin itch	47 (21)	11 (5)	**<0.001**
**Attitudes**			
N Participants who think they or any member of their family can get sick from water (%)	175 (78)	161 (72)	0.096
N Participants who think they can get diarrhea from drinking water (%)	102 (45)	121 (55)	0.064
N Participants who know water is safe to drink because (%)			
water is clear	211 (94)	214 (96)	0.404
it comes from the point-of-distribution	9 (4)	22 (9.9)	0.023
water is treated	43 (19)	72 (32)	0.002
it has chlorine smell or odor	31 (14)	43 (19)	0.149
N Participants who know water is not safe to drink because (%)			
it looks dirty	217 (96)	217 (97)	1
it makes sick	94 (42)	50 (22)	**<0.001**
it smells bad	85 (38)	84 (37)	1
it tastes bad	65 (29)	67 (30)	0.893
N Participants who believe the water they have collected today at the tapstand is safe to drink %)	140 (62)	166 (74)	**0.017**
N Participants who believe the water they have collected today at the tapstand is safe to drink because it has chlorine smell or odor (%)	16 (7)	48 (21)	**<0.001**
N Participants who think the water collected at the tapstand has a good taste (%)	114 (51)	77 (34)	**<0.001**
N Participants who think the water collected at the tapstand has a good smell (%)	101 (45)	101 (45)	**1**
N Participants who have detected any particular change in their water these past few weeks (%)	65 (29)	87 (39)	**<0.001**

**Dirty* being defined as being if the container has any visible dirt or green algae growing on the inside of the container.

Table S3: Summary of the number of samples collected during the study (n=2,768).

	Piped Water Evaluation		Trucked Water Evaluation	
	Baseline	*Endline*	*Baseline*	*Endline*
Point-of-distribution (T_0)	216 FRC<br>40 DBP	223 FRC	224 FRC	225 FRC
Point-of-consumption ($T_{<1h}$)	216 FRC<br>19 *E. coli*	223 FRC	224 FRC<br>15 *E. coli*	225 FRC
Point-of-consumption ($T_{3\text{-}24h}$)	189 FRC<br>19 *E. coli*<br>40 DBP	216 FRC	218 FRC<br>16 *E. coli*	220 FRC
Total	739	662	697	670

Table S4: Breakdown of FRC samples collected at the point of distribution at baseline and endline and included in the analysis, by distance from the treatment plan*.

Water System	Tapstand ID	# Samples (Baseline)	# Samples (Endline)
Piped Water Evaluation	T13	5 (2.9%)	12 (5.7%)
	T15	3 (1.7%)	12 (5.7%)
	T16	9 (5.2%)	12 (5.7%)
	T5	13 (7.5%)	12 (5.7%)
	T17	11 (6.3%)	13 (6.1%)
	T8	12 (6.9%)	15 (7.1%)
	T18	14 (8.0%)	14 (6.6%)
	T9	2 (1.1%)	6 (2.8%)
	T10	3 (1.7%)	5 (2.4%)
	T12	13 (7.5%)	14 (6.6%)
	T2	9 (5.2%)	14 (6.6%)
	T3	13 (7.5%)	15 (7.1%)
	T4	11 (6.3%)	15 (7.1%)
	T5	14 (8.0%)	14 (6.6%)
	T6	15 (8.6%)	14 (6.6%)
	T9	15 (8.6%)	13 (6.1%)
	T10	12 (6.9%)	12 (5.7%)
	Total	*174 (100%)*	*212 (100%)*
Trucked Water Evaluation	T01	147 (70.7%)	112 (52.6%)
	T02	147 (70.7%)	112 (52.6%)
	Total	*209 (100%)*	*213 (100%)*

*Please note the number of samples collected in tapstands in the piped water evaluation closest to the treatment plant (T13-T12) compared to those further (T2-T10) was not significantly different by Chi-squared test (p=0.113), and thus distance to treatment plant is not likely to be a reason for FRC differences seen.

Annex S1: Water Quality Testing and Survey: Initial Survey (Control and Intervention Groups)

[**Read:** Thank you for accepting to participate in the survey. Before getting started, I need to write down my name, the date, the location, and the point-of-distribution ID. Please give me a minute.]

a.	Interviewer Name	
b.	Date	
c.	Time	
d.	GPS coordinates	
e.	Point-of-distribution ID	
f.	HH ID	
g.	Are the security criteria met to follow the participant to his/her point-of-consumption?	Yes<br>No. Stop here.
h.	Selected study design	Before-after design. Go to Q1.<br>Difference-in-difference design. Go to Q1.<br>Matching design.
i.	Selected group	Intervention group. Go to Q1.<br>Control group.

[**Read:** I first need to ask you some general questions about you and your household to be sure I can include you in this study.]

j.	Since approximately how long do you live in this area?	
k.	How long do you take to come from the point-of-consumption to the point-of-distribution?	
l.	How many people live in your home?	
m.	*Give the definition/criteria of diarrhea and cholera.*<br>Has anyone in your home had diarrhea or cholera in the last week?	

➔ Based on the response, include or exclude the participant (if matches or not with the intervention group)

Part A: Distribution-point sampling

[**Read:** I need to take a water sample directly from the tap and to take some measurements.]

Q1.	[Observe. What is the type of the tap?]	
Q2.	[Record field collected parameters]	
Q3.	Time of sampling	[hh:mm]
Q4.	Air temperature	[°]
Q5.	Water temperature	[°]
Q6.	Turbidity	[NTU]
Q7.	Conductivity	[mg/L – ppm – ppt]
Q8.	pH	[-]
Q9.	FRC	[mg/L – ppm]
Q10.	TRC	[mg/L – ppm]

Part B: Observation on Water Collection and Transport

[**Read:** Now, you can collect your water. During this time, I need to write down some visual observations about your transport container(s).]

Q11.	[Observe. In how many transport containers is the water collected by the participant (including other people from the same household collecting water together)?]	1 container<br>2 containers<br>3 containers<br>Other: [number of containers]		
Q12.	[Observe. In what type of transport container was the water collected? Several answers possible if more than one container. If more than three containers, select the three biggest containers]	*Container 1*<br>Metal pot<br>Ceramic pot<br>Jerrycan<br>Plastic bottle<br>Bucket<br>Barrel<br>Other	*Container 2*<br>Metal pot<br>Ceramic pot<br>Jerrycan<br>Plastic bottle<br>Bucket<br>Barrel<br>Other	*Container 3*<br>Metal pot<br>Ceramic pot<br>Jerrycan<br>Plastic bottle<br>Bucket<br>Barrel<br>Other
Q13.	[Observe. Approximately how many liters is the transport container?]	[liters]	[liters]	[liters]
Q14.	[Observe. Is the transport container covered?<br>If there is any gap on the top of the container (e.g. no lid, broken on top, open cap), count it as uncovered]	Yes<br>No<br>Don't know	Yes<br>No<br>Don't know	Yes<br>No<br>Don't know
Q15.	[Observe. How large is the opening of the transport container? If the opening is smaller than the length of your finger, count is as small. If the opening is larger than the length of your hand, count is as large.]	Small<br>Medium<br>Large<br>Other	Small<br>Medium<br>Large<br>Other	Small<br>Medium<br>Large<br>Other
Q16.	[Observe. Is the transport container dirty? If the container has any visible dirt or green algae growing on the inside of the container, count it as dirty]	Yes<br>No<br>Don't know	Yes<br>No<br>Don't know	Yes<br>No<br>Don't know
Q17.	[Observe. Is the transport container translucent? If you can see the level of the water within the container, count it as translucent]	Yes<br>No<br>Don't know	Yes<br>No<br>Don't know	Yes<br>No<br>Don't know
Q18.	[Observe. Is there any hand contact with the water during collection?]	Yes<br>No<br>Don't know	Yes<br>No<br>Don't know	Yes<br>No<br>Don't know

[Accompany the participant to the point-of-consumption and note information on time and distance]

Q19.	Time when the participant leaves the point-of-distribution	[hh:mm]
Q20.	Time when the participant arrives at the point-of-consumption	[hh:mm]
Q21.	Approximate distance between the point-of-distribution and the point-of-consumption	[number of paces]

Part C: General Information

[**Read**: I will begin by asking you some general questions about your household]

Q22.	[Observe. What is the respondent's sex?]	Male<br>Female<br>Other
Q23.	How old are you?	[years]<br>Don't know
Q24.	Did you go to school?	Yes<br>No. Go to Q27.

		Don't know
Q25.	What is the highest grade you completed in school?	[grade]<br>Don't know
Q26.	Is the female head of house able to read and write?	Yes<br>No<br>No female head of house<br>Don't know
Q27.	Is the male head of house able to read and write?	Yes<br>No<br>No female head of house<br>Don't know
Q28.	How many people live in your home?	[persons]<br>Don't know
Q29.	How many beds are in your home?	[beds]<br>Don't know
Q30.	Since approximately how long do you live in this area?	[weeks]<br>[months]<br>[years]<br>Don't know
Q31.	*Give the definition/criteria of diarrhea and cholera.*<br>Has anyone in your home had diarrhea or cholera in the last week?	Yes<br>No<br>Don't know. Go to Q33.
Q32.	How many people have had diarrhea or cholera in the last week?	[persons]<br>Don't know
Q33.	Can you please show me where you usually wash your hands? [Observe location]	Dedicated space/handwashing facility<br>No dedicated space. Go to Q37.<br>Refuse. Go to Q37.<br>Don't know. Go to Q37.<br>Other
Q34.	Approximate distance between handwashing facility and the point-of-consumption	[number of paces]<br>Don't know
Q35.	Can you please show me what you use to wash your hands? [Observe if soap is present]	Soap present<br>Soap absent<br>Refuse<br>Other
Q36.	[Observe if water is present]	Running water<br>Water in container<br>No water<br>Other
Q37.	Can you please show me where you usually use the bathroom? [Observe location]	Private latrine<br>Shared latrine<br>No latrine. Go to Q39.<br>Refuse. Go to Q39.<br>Don't know. Go to Q39.<br>Other
Q38.	Approximate distance between latrine and point-of-consumption	[number of paces]<br>Don't know

Part D: Water Collection and Transport Practices

Q39.	Approximately how long ago did you obtain the transport container(s)?	[weeks]	[weeks]	[weeks]

		[months]<br>[years]<br>Don’t know	[months]<br>[years]<br>Don’t know	[months]<br>[years]<br>Don’t know
Q40.	Do you clean the transport container(s)?	Yes<br>No. Go to Q43.<br>Don’t know. Go to Q43.		
Q41.	How often do you clean the transport container(s)?	Daily<br>Weekly<br>Rarely<br>Don’t know<br>Other		
Q42.	What do you use to clean the transport container? [Multiple answers possible]	Water from the point-of-distribution<br>Other water source<br>Soap<br>Bleach<br>Rocks/sand<br>Don’t know<br>Other		
Q43.	Where do you store this container when empty?	Inside<br>Outside<br>Don’t know<br>Other		
Q44.	Who collects water in your household?	Me<br>Don’t know<br>Other		
Q45.	Is it always the same person who collects water in your household?	Yes<br>No<br>Don’t know		
Q46.	Do you always collect water from a point-of-distribution?	Yes. Go to Q49.<br>No<br>Don’t know		
Q47.	When or why don’t you always collect water from a point-of-distribution? [Multiple answers possible]	Too far<br>Unsafe<br>Don’t know<br>Other		
Q48.	Apart from a point-of-distribution, where do you also collect water? [Multiple answers possible]	Unprotected source<br>Protected source<br>Don’t know<br>Other		
Q49.	Do you always collect water from the same point-of-distribution?	Yes. Go to Q51.<br>No<br>Don’t know		
Q50.	When or why don’t you always collect water from the same point-of-distribution? [Multiple answers possible]	Too far<br>Too crowded<br>Don’t know<br>Other		
Q51.	How many times a day do you or a member of your household go to collect water from the point-of-distribution?	<1<br>1<br>2<br>Don’t know<br>Other		

Q52.	When do you go to collect water from the point-of-distribution? [Multiple answers possible]	Morning<br>Afternoon<br>Evening<br>Don't know<br>Other
Q53.	For approximately how many hours do you store the collected water?	A Few hours (< 6 hours)<br>A single day (< 12 hours)<br>From afternoon/overnight until next morning (~18 hours)<br>From morning until next morning (~24 hours)<br>Don't know
Q54.	What do you use the water you've collected at the point-of-distribution? [Multiple answers possible]	Drinking<br>Cleaning dishes<br>Washing hands<br>Cleaning house<br>Washing food<br>Bathing<br>Don't know<br>Other

Part E: Water Storage Practices

[When you are at the point-of-consumption, ask the participant to do with the water as (s)he would normally do.]

Q55.	[Observe. Is the water of interest transferred into another storage container?]	Yes.<br>No. Go to Q69.		
Q56.	[Observe. In how many storage containers is the water poured into?]	1 storage container<br>2 storage containers<br>3 storage containers<br>Other: [number of containers]		
Q57.	[Observe each storage container that the water is poured into. Is there already water present in those containers? Several answers possible if more than one container. If more than three storage containers, select the three biggest containers]	*Storage 1*<br>Yes<br>No<br>Don't know	*Storage 2*<br>Yes<br>No<br>Don't know	*Storage 3*<br>Yes<br>No<br>Don't know
Q58.	[Observe. In what type of storage container is the water stored?]	Metal pot<br>Ceramic pot<br>Jerrycan<br>Plastic bottle<br>Bucket<br>Barrel<br>Other	Metal pot<br>Ceramic pot<br>Jerrycan<br>Plastic bottle<br>Bucket<br>Barrel<br>Other	Metal pot<br>Ceramic pot<br>Jerrycan<br>Plastic bottle<br>Bucket<br>Barrel<br>Other
Q59.	[Observe. Approximately how many liters is the storage container?]	[liters]	[liters]	[liters]
Q60.	Observe. Is the storage container covered?<br>If there is any gap on the top of the container (e.g. no lid, broken on top, open cap), count it as uncovered]	Yes<br>No<br>Don't know	Yes<br>No<br>Don't know	Yes<br>No<br>Don't know
Q61.	[Observe. How large is the opening of the storage container? If the opening is smaller than the length of your finger, count is as small. If the opening is larger than the length of your hand, count is as large.]	Small<br>Medium<br>Large<br>Other	Small<br>Medium<br>Large<br>Other	Small<br>Medium<br>Large<br>Other

Q62.	[Observe. Is the storage container dirty? If the container has any visible dirt or green algae growing on the inside of the container, count it as dirty]	Yes<br>No<br>Don't know	Yes<br>No<br>Don't know	Yes<br>No<br>Don't know
Q63.	[Observe. Is the storage container translucent? If you can see the level of the water within the container, count it as translucent]	Yes<br>No<br>Don't know	Yes<br>No<br>Don't know	Yes<br>No<br>Don't know
Q64.	[Observe. Is there any hand contact with the water during usage?]	Yes<br>No<br>Don't know	Yes<br>No<br>Don't know	Yes<br>No<br>Don't know

Q65.	Approximately how long ago did you obtain the storage container?	[weeks]<br>[months]<br>[years]<br>Don't know	[weeks]<br>[months]<br>[years]<br>Don't know	[weeks]<br>[months]<br>[years]<br>Don't know
Q66.	Do you clean the storage container?	Yes<br>No. Go to Q69.<br>Don't know. Go to Q69.		
Q67.	How often do you clean the storage container?	Daily<br>Weekly<br>Rarely<br>Don't know<br>Other		
Q68.	What do you use to clean the storage container?<br>[Multiple answers possible]	Water from the point-of-distribution<br>Other water source<br>Soap<br>Bleach<br>Rocks/sand<br>Don't know<br>Other		
Q69.	Where do you store this container when there is water?	Inside<br>Outside<br>Don't know<br>Other		
Q70.	Where do you store this container when empty?	Inside<br>Outside<br>Don't know<br>Other		

Part F: Consumption-point sampling (I)

[**Read**: I will collect a sample of the water in the water container. I will take [quantity] of water from your container to do the testing.]

Q71.	Can you please give me a cup of water that you would drink?	Yes<br>No/refuse. [End. Go to Q101]
Q72.	[Which container do you sample?]	Transport container 1<br>Transport container 2<br>Transport container 3<br>Storage container 1<br>Storage container 2<br>Storage container 3<br>Other

Q73.	[Record field collected parameters]	
Q74.	Time of sampling	[hh:mm]
Q75.	Air temperature	[°]
Q76.	Water temperature	[°]
Q77.	Turbidity	[NTU]
Q78.	Conductivity	[mg/L – ppm - ppt]
Q79.	pH	[-]
Q80.	FRC	[mg/L – ppm]
Q81.	TRC	[mg/L – ppm]
Q82.	Can I take a picture of the container?	Yes. Take a picture.<br>No. Do not take a picture.
Q83.	Can I mark the container I sampled?	Yes. Mark the container.<br>No/refuse. Do not mark the container.

Part G: Water Treatment Knowledge

[**Read**: I will ask you questions about your water. This is the last part of the survey for today]

Q84.	Do you think you or any member of your family can get sick from water?	Yes<br>No<br>Don't know
Q85.	What kind of sickness can you or any member of your family get from drinking water?<br>[Probe] Any more?<br>[Multiple answers possible]	Diarrhea<br>Vomiting<br>Stomachache<br>Fever<br>Cholera<br>Dehydration<br>Headache<br>Influenza<br>General pain<br>Parasites<br>Don't know<br>Other
Q86.	How do you know if your water is safe to drink?<br>[Probe]Any other reason?<br>[Multiple answers possible]	Water is clear<br>No bacteria<br>It comes from the point-of-distribution<br>Water is treated<br>Used for a long time and not sick<br>Has chlorine smell or odor<br>Don't know<br>Other
Q87.	How might you know if your water is not safe to drink?<br>[Probe]Any other reason?<br>[Multiple answers possible]	Looks dirty<br>Makes sick<br>Bad source<br>Bad smell<br>Bad taste<br>Stored in open container<br>Don't know<br>Other
Q88.	Do you believe that the water you have collected today at the point-of-distribution is safe to drink?	Yes. Go to Q90.<br>No.<br>Don't know. Go to Q91.

<table>
<tr><td>Q89.</td><td>Why do you think the water you have collected today at the point-of-distribution is not safe to drink? [Multiple answers possible]</td><td colspan="3">Looks dirty
Makes sick
Bad source
Bad smell
Bad taste
Stored in open container
Don’t know
Other</td></tr>
<tr><td>Q90.</td><td>Why do you think the water you have collected today at the point-of-distribution is safe to drink? [Multiple answers possible]</td><td colspan="3">Water is clear
No bacteria
It comes from the point-of-distribution
Water is treated
Used for a long time and not sick
Don’t know
Other</td></tr>
<tr><td rowspan="8">Q91.</td><td rowspan="8">Please tell me about any methods you know of for making your water safe to drink.
[Circle Yes or No for each]
[Probe] Do you know any more ways to make your water safe to drink?

Now I would like to know if you have ever used any of those methods to treat your drinking water, and how often.
You said you know about [boiling].
Do you use [boiling] ?
How often do you use [boiling]?</td><td>Method</td><td>Known</td><td>Used</td></tr>
<tr><td>Boiling</td><td>Yes No</td><td>No Rarely Often Always</td></tr>
<tr><td>Liquid chlorine</td><td>Yes No</td><td>No Rarely Often Always</td></tr>
<tr><td>Cloth filter</td><td>Yes No</td><td>No Rarely Often Always</td></tr>
<tr><td>Other filter</td><td>Yes No</td><td>No Rarely Often Always</td></tr>
<tr><td>Tablets</td><td>Yes No</td><td>No Rarely Often Always</td></tr>
<tr><td>Other:</td><td>Yes No</td><td>No Rarely Often Always</td></tr>
<tr><td colspan="3">Don’t know</td></tr>
<tr><td>Q92.</td><td>Have you received any messaging about the chlorinated water?</td><td colspan="3">Yes
No. Go to Q97.
Don’t know. Go to Q97.</td></tr>
<tr><td>Q93.</td><td>What have you learned?</td><td colspan="3">Cover container
Wash container
Don’t know
Other</td></tr>
<tr><td>Q94.</td><td>What do you like about having chlorine in your drinking water?</td><td colspan="3">Makes it safe
Good taste
Smell clean
People are less sick
Don’t know
Other</td></tr>
<tr><td>Q95.</td><td>What do you dislike about having chlorine in your drinking water?</td><td colspan="3">Chemicals are bad
Bad taste
Bad smell
Don’t know
Other</td></tr>
<tr><td>Q96.</td><td>Do you know if the water you have collected today at the point-of-distribution is chlorinated?</td><td colspan="3">Yes
No
Don’t know</td></tr>
</table>

Q97.	Do you think the water collected at the point-of-distribution has a good taste?	Yes<br>No<br>Don’t know
Q98.	Do you think the water collected at the point-of-distribution has a good smell?	Yes<br>No<br>Don’t know
Q99.	Have you detected any particular change in your water these past few weeks?	Yes<br>No. Go to Q101.<br>Don’t know. Go to Q101.
Q100.	What is the change in your water you have detected? [Multiple answers possible]	Water taste<br>Water smell<br>Don’t know<br>Other

Q101.	Is there something else you would like to share?	
Q102.	Report end time	[hh:mm]

[**Read**: Thank you for your time. I will come back [Time]. Meanwhile please continue to use the water as you normally would, including using it all up if you need to].

Annex S2: Water Quality Testing and Survey: Follow-up Survey (Control and Intervention Groups)

[**Read**: Thank you for accepting to participate in the survey. Before getting started, I need to write down my name, the date, time, location, and the HH ID. Please give me a minute.]

n.	Interviewer Name	
o.	Date	
p.	Time	
q.	Location ID	
r.	HH ID	

Part H: Available Drinking Water

Q103.	Were you or someone in your family interviewed by our team previously?	Yes. Go to Q109.<br>No.<br>Don't know [End?]
Q104.	Is the person we interviewed previously available to speak with us? [If they are available, please complete the survey with them]	Yes. Go to Q109.<br>No<br>Don't know.
Q105.	[Observe. What is the respondent's sex?]	Male<br>Female<br>Other
Q106.	How old are you?	[years]<br>Don't know
Q107.	Did you go to school?	Yes<br>No. Go to Q109.<br>Don't know. Go to Q109.
Q108.	What is the highest grade you completed in school?	[grade]<br>Don't know

Q109.	Do you have any water that you collected during our previous visit's?	Yes<br>No water. [End. Go to Q139]<br>Don't know. [End. Go to Q139]
Q110.	May I see the water that was collected from the point-of-distribution yesterday?	Yes<br>No/Refuse. [End. Go to Q139]<br>No water. [End. Go to Q139]
Q111.	[Observe. Approximately what is the amount of water left?]	[liters]<br>Don't know
Q112.	[Observe. Is there still a mark on the container that was sampled during the previous visit?]	Yes. Go to Q120.<br>No

Part I: Observation on Water Storage Practices

Q113.	[Observe. In what type of container is the water stored?]	Metal pot<br>Ceramic pot<br>Jerrycan<br>Plastic bottle<br>Bucket<br>Barrel<br>Other

Q114.	[Observe. Approximately how many liters is storage the container?]	[liters]
Q115.	Observe. Is the storage container covered?<br>If there is any gap on the top of the container (e.g. no lid, broken on top, open cap), count it as uncovered]	Yes<br>No<br>Don't know
Q116.	[Observe. How large is the opening of the storage container? If the opening is smaller than the length of your finger, count is as small. If the opening is larger than the length of your hand, count is as large.]	Small<br>Medium<br>Large<br>Other
Q117.	[Observe. Is the storage container dirty? If the container has any visible dirt or green algae growing on the inside of the container, count it as dirty]	Yes<br>No<br>Don't know
Q118.	[Observe. Is the storage container translucent? If you can see the level of the water within the container, count it as translucent]	Yes<br>No<br>Don't know
Q119.	[Observe. Where is the storage container stored?]	Inside<br>Outside<br>Don't know<br>Other

Part J: Water Treatment Practices

Q120.	Have you added other water to this container since our last visit?	Yes<br>No<br>Don't know
Q121.	Did someone at the house treat this water in any way since our visit yesterday?	Yes<br>No. Go to Q124.<br>Don't know. Go to Q124.
Q122.	How was the water treated? [Multiple answers possible]	Boiled<br>Tablets<br>Cloth filter<br>Other filter<br>Liquid chlorine<br>Don't know<br>Other
Q123.	Approximately how long ago was the water treated?	[minutes]<br>[hours]<br>Don't know

Part K: Water Sampling

[**Read**: I will collect a sample of the water you have collected from the point-of-distribution yesterday. I will take [quantity] of water from the container I sampled yesterday to do the testing.]

Q124.	Can you please give me a cup of water that you would drink?	Yes<br>No/refuse. [End. Go to Q137]
Q125.	[Are you able to sample the same container you sampled yesterday?]	Yes<br>No<br>Don't know
Q126.	[Record field collected parameters]	
Q127.	Time of sampling	[hh:mm]
Q128.	Air temperature	[°]

Q129.	Water temperature	[°]
Q130.	Turbidity	[NTU]
Q131.	Conductivity	[mg/L – ppm - ppt]
Q132.	pH	[-]
Q133.	FRC	[mg/L – ppm]
Q134.	TRC	[mg/L – ppm]
Q135.	[If selected participant, take a sample in a Whirl-Pak bag]	[Sample ID]
Q136.	Can I take a picture of the container?	Yes. Take a picture.<br>No. Do not take a picture.

Q137.	Is there something else you would like to share?	
Q138.	Report end time	[hh:mm]
